# Therapeutic Perspectives of 8-Prenylnaringenin, a Potent Phytoestrogen from Hops

**DOI:** 10.3390/molecules23030660

**Published:** 2018-03-15

**Authors:** Kateřina Štulíková, Marcel Karabín, Jakub Nešpor, Pavel Dostálek

**Affiliations:** Department of Biotechnology, University of Chemistry and Technology, Prague, Technická 5, 166 28 Prague 6, Czech Republic; marcel.karabin@vscht.cz (M.K.); nesporj@vscht.cz (J.N.); pavel.dostalek@vscht.cz (P.D.)

**Keywords:** 8-prenylnaringenin, menopause, hops, phytoestrogens

## Abstract

Hop (*Humulus lupulus* L.), as a key ingredient for beer brewing, is also a source of many biologically active molecules. A notable compound, 8-prenylnaringenin (8-PN), structurally belonging to the group of prenylated flavonoids, was shown to be a potent phytoestrogen, and thus, became the topic of active research. Here, we overview the pharmacological properties of 8-PN and its therapeutic opportunities. Due to its estrogenic effects, administration of 8-PN represents a novel therapeutic approach to the treatment of menopausal and post-menopausal symptoms that occur as a consequence of a progressive decline in hormone levels in women. Application of 8-PN in the treatment of menopause has been clinically examined with promising results. Other activities that have already been assessed include the potential to prevent bone-resorption or inhibition of tumor growth. On the other hand, the use of phytoestrogens is frequently questioned regarding possible adverse effects associated with long-term consumption. In conclusion, we emphasize the implications of using 8-PN in future treatments of menopausal and post-menopausal symptoms, including the need for precise evidence and further investigations to define the safety risks related to its therapeutic use.

## 1. Introduction

Hop (*Humulus lupulus* L., Cannabaceae) is primarily known for the aroma and taste it gives beer, in particular, as the essential determinant of bitterness. However, its uses have become more diverse in recent years. It is appreciated in phytotherapy, for its sedative, digestive, anti-inflammatory, and other effects [1]. Since hop is a source of miscellaneous compounds, e.g. essential oils, bitter acids, and polyphenols, and many of them exert biological activities, interest in its potential therapeutic applications has been growing [2]. However, current legislation prevents hop from being classified as a herbal medicine, because there is insufficient evidence for its medicinal use over the required period of time. Branding it as a “nutritional supplement” could provide an alternative route for the introduction of hop-based remedies to the pharmaceutical market, but prior to that, in order to meet the requirements for this classification, its “health claim” must be approved by the European Food Safety Authority. Currently, the claim that hop relieves menopausal symptoms is awaiting approval [3]. Beneficial properties of prenylated flavonoids, a specific group of hop biologically active compounds, have become a topic of scientific interest. They are frequently reported for their estrogenic activity [4,5,6]. The estrogenic potential of prenylated flavonoids from hops represents a new therapeutic solution to alleviate menopausal and post-menopausal symptoms in women [7]. In particular, 8-prenylnaringenin (8-PN), is considered to be the most potent phytoestrogen thus far discovered [8]. Menopause is characterized by a progressive decline in estrogenic hormone levels, and is often associated with unpleasant symptoms, such as hot flashes, mood swings, lack of energy, joint soreness, and increased risk of osteoporosis [9]. The current therapeutic approach is mainly hormone replacement therapy (HRT). However, the results of clinical trials of HRT have been correlated with certain adverse health effects, including increased risk of thrombosis and increased occurrence of ovarian and breast cancer [10,11,12]. Thus, the demand for alternative therapeutic solutions has been emerging. A few clinical trials have already been conducted to evaluate the efficiency of hop-based preparations to relieve menopausal discomforts [13,14,15]. Further investigations have focused on assessments of anticancer properties and the potential to prevent osteoporosis during menopause [16,17,18,19,20,21].

In support of hop’s “health claim” for approval, we present a summary of current literature to explain the mechanisms of estrogenic activity of 8-PN, and its pharmacokinetics and biotransformation. Our review underlines the therapeutic potential of the natural phytoestrogen from hops.

## 2. Prenylated Flavonoids

Prenylated flavonoids represent a group of secondary metabolites found in hops (*Humulus lupulus* L.). Examples of other plant families characterized by the production of prenylated flavonoids are Leguminosae, Moraceae, Guttiferae, Umbelliferae, and Rutaceae [22]. In contrast to the majority of hop flavonoids that are found in the seeds and bracts inside hop cones, prenylated flavonoids are gathered in lupulin glands, together with resins and essential oils [23]. In general, flavonoids are plant metabolites with the basic core structure of flavane (2-phenyl-benzo-γ-pyrane), structurally described as two benzene rings (A and B) linked together by a pyrane ring (C) [17]. In addition, prenylated flavonoids possess a prenyl group attached to the flavane nucleus [24]. Furthermore, these constituents are divided according to their chemical structure into prenylated chalcones (xanthohumol, desmethylxanthohumol) and prenylated flavanones (6-prenylnaringenin, 8-PN) [23].

**Figure 1:**
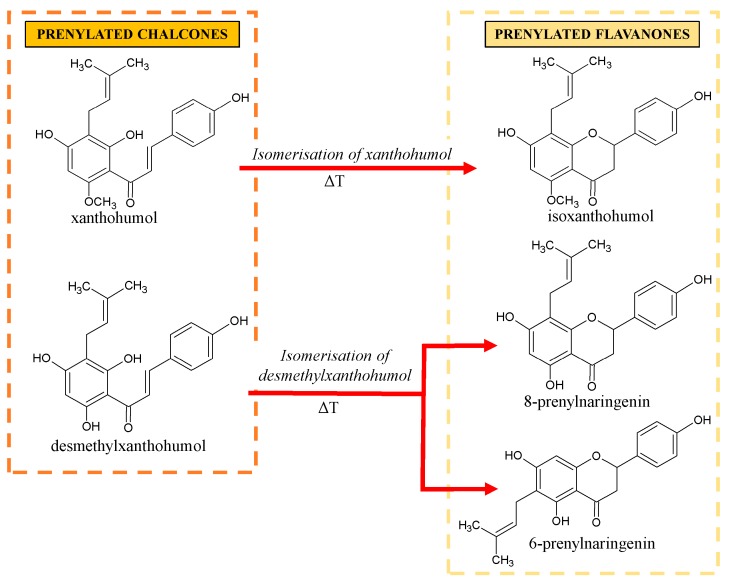
Prenylated flavonoids from hops.

While the content of xanthohumol in dry hop matter can be up to 1%, the content of 8-PN is more than 10 times lower [25]. The exact amount of each constituent is difficult to quantify precisely, because chalcones can undergo isomerization, resulting in the formation of isoanalogues, e.g., conversion of xanthohumol in isoxanthohumol upon thermal treatment, and desmethylxanthohumol isomerizes into a racemic mixture of 6- and 8-prenylnaringenin [1] (Figure 1).

Extraction of prenylated flavonoids from plant sources involves several steps to assure the purity of the product. Another critical point is to guarantee that metabolites do not undergo any structural modifications and do not degrade upon the extraction process [26]. Efficient methods suggested so far are ultrasound-assisted extraction, supercritical fluid extraction, or microwave-assisted extraction. To quantify the content of individual prenylated flavonoids and fragments, liquid chromatography-tandem mass spectrometry has been shown to be the most suitable method [27].

## 3. Mechanism of Action, Pharmacokinetics, and Biotransformation

Phytoestrogens are a general term for compounds of various structures, originating in plant sources and mimicking or modulating the action of estrogenic hormones, due to their ability to interact with estrogenic receptors (ER) [5]. Apart from the ER-activating pathway, phytoestrogens can also exhibit their effects through non-genomic mechanisms, via interactions with cell surface receptors or by epigenetic mechanisms [28].

The estrogenic potential of certain plants was first reported in the 1940s from an observation of sheep that suffered reproductive disorders as a consequence of a diet mostly based on clover (*Trifolium subterraneum*, Fabaceae). The group of symptoms occurring in these sheep, abnormal lactation and morphological changes in reproductive organs, was defined as clover disease. Furthermore, female hop-pickers suffered from disturbances in their menstrual cycles during the hop harvests, and this phenomenon led to the assumption that hops exerted estrogenic activities [29]. At that time, hop baths were applied as a traditional therapy for several gynecological difficulties. The active substance, 8-PN, which is responsible for the estrogenic effect, was identified in 1999 by Milligan [30].

As well as prenylated flavonoids, some other groups of plant secondary metabolites also exhibit estrogenic effects. From the point of view of chemical structures, “plant estrogens“, phytoestrogens, comprise isoflavones, lignans, and coumestans [3]. The intake of phytoestrogens derives from dietary sources; genistein and daidzein, examples from the isoflavone group, are present in soy, lignans are formed from various seed and vegetable precursors by intestinal bacteria, and the main coumestan, coumestrol, is present in peas, beans, and sprouts [31].

Estrogenic hormones are primarily involved in regulation of the reproductive and central neural systems. They also maintain bone density, stimulate growth, regulate water retention, and influence blood coagulation. The extent of estrogenic actions of certain compounds is generally defined by their affinities to receptors, and the presence of transcriptional co-activators or promoter corepressors [32]. Two types of estrogen receptors (ER) are distinguishable in humans; ERα is mainly expressed in the endometrium, ovarian stroma, bones, and in the mammary gland, while ERβ is prevalent in adipose tissue, endothelial cells, brain, kidneys, and in the prostate gland [33,34]. Substrates binding to ER usually have a preferential affinity to one receptor type. In this regard, the majority of phytoestrogens show a preference for ERβ [6], while 8-PN predominantly binds to ERα, with approximately 100 times higher affinity compared to genistein [35,36]. 8-PN is categorized as a natural selective estrogen receptor modulator (SERM). The term selective refers to compounds that act as ER agonists in some tissues, and as antagonists in others [37]. An example of such a compound is the drug tamoxifen, which is used in the treatment of breast cancer, acting as an antagonist in the breast, suppressing transcription via binding to ERα, and simultaneously acting as an agonist on ERβ in the uterus [38]. In drug design, it is possible to adjust the preference of a compound to bind selectively either to ERα or ERβ [39]. The significance of the prenyl group for interacting with ER is crucial. The 8- substituent induces conformational changes in the receptor by binding of ligands, and therefore, the chain length and branching of the group have key impacts on transcriptional outcomes [40]. Furthermore, the estrogenicity of 8-PN is significantly enhanced by the 8-prenyl group compared to naringenin, as has been shown in experiments using three ER model assays [41]. The prenyl group increases the hydrophobicity of the molecule, and thereby facilitates interactions with biological membranes and lipophilic proteins [42]. As a result, prenylation increases uptake by intestinal cells. On the other hand, the prenyl group diminishes efflux by adenosine triphosphate-binding cassette transporters, such as the multidrug-resistant protein 2 and the breast cancer-resistant protein (BCRP). Consequently, prenylation reduces the bioavailability of 8-PN in comparison to non-prenylated naringenin [8]. Inhibition of the BCRP transporter by 8-PN has been further confirmed by Tan et al. [43].

The pharmacokinetic profile of 8-PN was described through surveillance of 24 postmenopausal women after oral administration of the drug. The maximum plasma concentration was reached in 1–1.5 h, and a second peak occurred after 7–10 h as a result of enterohepatic recirculation [44]. 8-PN does not undergo significant conversion during phase I metabolism, in contrast to estradiol or ethinyl estradiol [45]. During phase 2, glucuronidation and sulfation are the predominant reactions. In addition, broad differences in the maximum plasma concentrations were observed between individuals, most plausibly due to polymorphisms in the biotransformation enzymes involved in metabolism [46]. Notably, a 750 mg dose of 8-PN decreased the concentration of luteinizing hormone in postmenopausal women, suggesting that 8-PN could penetrate the blood-brain barrier [44]. However, in a clinical trial with 16 volunteers, the levels of luteinizing hormone were not affected by administration of a single oral dose containing 500 mg of 8-PN [46]. In another pharmacokinetic study, a standardized hop extract was administered to 5 postmenopausal women to measure the bioavailability of active compounds, and to observe whether metabolism of the mixture of active compounds influenced the bioavailability of any of them. No effects were detected on sex hormone levels, nor on blood clotting, and the maximum concentrations in the serum were in a similar range as described previously [47]. The linear relationship between dose and maximum blood concentration was confirmed. After absorption from the gastrointestinal tract, metabolism in the liver includes oxidation (hydroxylation) of the prenyl group. The prenyl substituent can also undergo epoxidation followed by cyclization [48]. It has been shown that certain microorganisms can convert 8-PN into oxygenated, glucosylated, or acyl-glucosylated metabolites via microbial transformation [49]. In humans, metabolites of 8-PN are primarily eliminated by biliary secretion into feces, since no significant amount of the metabolites were detected in the urine [47].

In the distal colon, up to the 80% of isoxanthohumol undergoes demethylation and is converted into 8-PN by intestinal microbiota [50]. This has to be taken into account because the intestinal conversion from isoxanthohumol can subsequently contribute to overall estrogenic activity, where hop extracts or hop preparations are used in comparison with the application of pure 8-PN [46].

## 4. Menopause Therapy

**Scheme 1:**
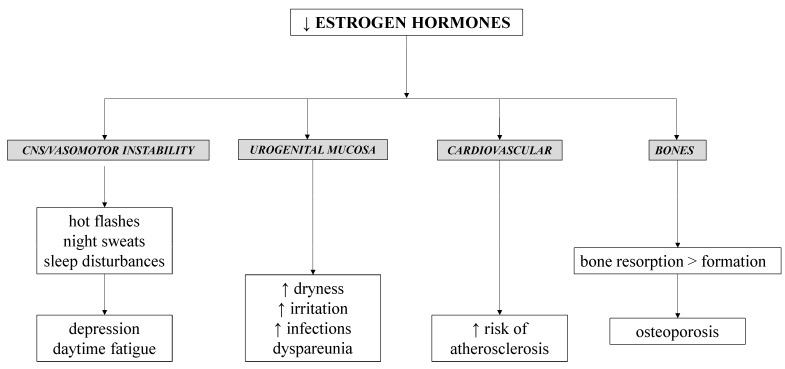
Menopause symptoms as a result of progressive decline of estrogens.

Phytoestrogens are the subject of scientific investigation, due to their potential as alternative therapeutics for health problems associated with altered levels of sex hormones. Such an example is menopause, a stage in women´s lives characterized by withdrawal of the menstrual period as a result of a natural progressive decline in estrogen production [51]. Menopause is accompanied by a number of symptoms, of which hot flashes and night sweats are the most frequent reasons for seeking medical support [52]. These symptoms are the result of changes in thermoregulation due to diminished estrogen levels. Other non-vasomotor symptoms are related to changes in the levels of neurotransmitters, mainly serotonins, causing depression, insomnia, irritability, loss of libido, and difficulties in concentrating. Other features are connected with lowered levels of estrogens and androgens in peripheral tissues, and involve vaginal dryness, irritation, and itching, commonly designated as the genitourinary syndrome of menopause [53]. Long-term consequences of the lack of estrogenic stimulation are increased risk of atherosclerosis, decrease in bone density, and progressive development of osteoporosis [28] (Scheme 1).

The conventional therapeutic approach to relieve menopausal symptoms is represented by HRT, consisting of hormonal substitution by the application of estrogens or estrogens in combination with progestins, available in several dosage forms, e.g., oral, transdermal, or intrauterine [54]. Nonetheless, a large clinical trial carried out by the Woman´s Health Initiative (WHI) in 2002 has revealed an increased risk of breast cancer, and a risk of thromboembolism in post-menopausal women as a consequence of HRT. Awareness of estimating the correct risk–benefit ratio for each patient has been highlighted, and therapeutic approaches have been individualized in terms of dosing, type of preparation, or ethnicity to diminish the risks of adverse effects [3]. Consequently, the frequency of HRT applications has declined due to the outcomes of these trials. However, the approaches used by WHI to process the data and interpret the results have been questioned, and the benefits associated with the use of HRT in menopausal and post-menopausal women, such as cardioprotective effects, prevention of fractures and control of vasomotor symptoms, are now being emphasized [55,56,57]. Despite this, complementary and alternative therapeutic approaches are in demand [53,58,59,60]. Therefore, many women choose nutritional supplements containing phytoestrogens from herbal sources as an alternative to HRT [61]. Common sources of phytoestrogens traditionally used for the relief of menopausal symptoms are black cohosh, red clover, and ginseng [7].

The effects of 8-PN and its potency to alleviate menopause-associated problems have been tested in several trials. In an animal model of menopausal hot flashes, represented by a method of measuring the tail skin temperature (TST) of ovariectomized rats upon administration of estrogenic compounds, the effects of 8-PN to restore the normal TST after subcutaneous or oral dosage were confirmed. This suggests that 8-PN could also be used to relieve hot flashes in women [62].

In a prospective, double blind, placebo-controlled study, the application of standardized hop extract over a period of 12 weeks was tested for its ability to alleviate menopausal discomforts in 67 menopausal women. The dosage of hop extract was 100 µg/day or 250 µg/day [13]. Outcomes of the survey were evaluated by the Kupperman index (KI), which is a scale indicating the severity of the most common menopausal symptoms [63]. In the 100 µg/day dose group there was a significant decrease in KI compared to the placebo, demonstrating the potential of the hop actives to relieve menopausal symptoms. Regarding the rating score for hot flashes particularly, efficacy was significant in both groups compared to the placebo. However, no dose–response linearity was detected, because higher doses were less active than lower ones [13]. In another study by the same research group, standardized hop extract (dose of 10 µg of 8-PN per day) was applied to a group of 36 menopausal women for a period of 8 weeks, and then for another 8 weeks, the treatment method was switched from active treatment to placebo, and vice versa. After 16 weeks, administration of the active dose after placebo treatment led to a reduction in menopausal symptoms, whereas administration of placebo after the active treatment increased the symptoms according to KI values. Interestingly, both the active drug, as well as the placebo group showed improvement, which can be explained by the fact that menopausal trials are typically sensitive to placebo effects, especially during short-term treatments [15].

Another placebo-controlled trial conducted in 2013 involved 120 women in the age range of 40–60 years. Some of them were premenopausal patients experiencing hot flashes with less than 12 menstrual bleedings within the past 12 months, and the selection parameter for post-menopausal women was a minimum of one year and maximum of 5 years after the last period. Patients were administered 500 mg dried hop tablets containing 100 µg of phytoestrogens. The exact content of 8-PN in the tablets was not specified in the report. The intervention lasted for 90 days. Severities of individual symptoms were evaluated through a checklist at the end of weeks 4, 8, and 12, and in all groups of symptoms, there was a dramatic difference between the hop group and the placebo. Overall, a significant efficacy of hop to ease early menopausal symptoms was observed in this survey, and no adverse effects related to the treatment were identified [14].

## 5. Prevention of Osteoporosis

Because osteoporosis is another unfavorable condition associated with menopause, the potential of 8-PN to prevent the loss of bone mass has been assessed [64,65]. In contrast to most other phytoestrogens that have been investigated for the treatment of menopause-related osteoporosis with disappointing results, 8-PN showed more favorable outcomes due to its preferential binding to ERα, which is predominant in bone tissue [66]. So far, the potential of 8-PN to prevent osteoporosis has been assessed using in vitro and in vivo models, but unfortunately, no clinical trials in humans have yet been conducted.

**Figure 2:**
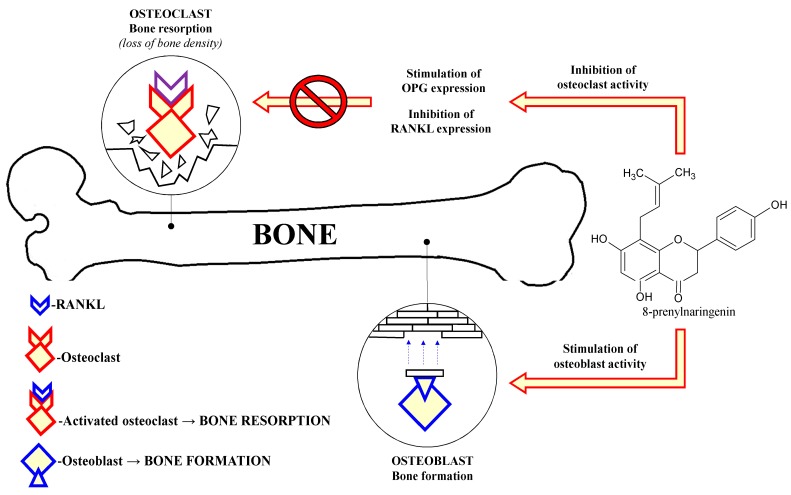
Role of 8-prenylnaringenin (8-PN) in prevention of osteoporosis.

The ability of 8-PN to promote differentiation of osteoblasts was compared with naringenin in order to confirm that the presence of the prenyl group was crucial to the anti-osteoporotic mechanism. 8-PN showed a stronger capability to induce the expression of osteoprotegerin (OPG), an osteoclastogenesis inhibitor, and to increase levels of osteoblast differentiation markers in cultured rat calvarial osteoblasts compared to naringenin. Moreover, in rabbit bone marrow cells, 8-PN inhibited the formation and induced apoptosis of osteoclasts to a greater extent than naringenin, confirming the essential role of the prenyl group in bone protective activities [67]. The effects of 8-PN on bone-metabolism were shown to be mediated by the ERα signaling pathway, and the intensities of responses in osteoblast and osteoclast cell lines were weaker in comparison with the effect of 17β-estradiol, but stronger than in the case of genistein and daidzein. 8-PN was applied to the MC3T3-E1 osteoblast cell line, where it enhanced differentiation and maturation, and also inhibited the differentiation of the RAW264.7 osteoclast cell line. Furthermore, 8-PN inhibited the expression of receptor activator of nuclear factor-κB ligand (RANKL), and led to increased expression of OPG [68]. Suggested mechanisms of 8-PN in prevention of osteoporosis are illustrated in Figure 2. A study in vivo, in ovariectomized rats (a model to mimic the natural decline of sexual hormones) that were administered, orally, a standardized hop extract for 8 weeks, was performed to investigate the effects of hops on preventing bone resorption and to evaluate the estrogenic mechanism of action in triggering proliferation in the endometrium. Analysis of vertebrae and tibia revealed a decline in the number of osteoclasts in the tibia, but in the lumbar vertebrae, the thickness and the number of trabeculae were even below the values of the ovariectomized control group [21].

## 6. Anticancer and Other Miscellaneous Effects of 8-PN

Hop phytoestrogens are known to exhibit anticancer activities [19]. Generally, prenylated chalcones are suggested to act against cancer cells by induction of autophagy or by modulating the cell cycle, and the efficacy in suppressing the growth of tumor cells was tested in a number of models in vitro [17]. 8-PN showed cytotoxic potential in the U-118 MG cancer cell line, and the accumulation of the compound was higher in cancer cells compared to human fibroblast cells. This property could be further utilized in the development of new anticancer drugs [18]. A significant, dose-dependent inhibition of proliferation was observed in PC-3 human prostate cancer cells and UO.31 human renal carcinoma cells after exposure to 8-PN [20]. Due to its estrogenic potency, 8-PN has been specifically tested for its activities against hormone-dependent breast tumors. In MCF-10A, a human breast cancer cell line, the drug modulated the metabolic pathways of estradiol conversion into cancer-promoting metabolites, and thereby inhibited malignant transformation [69,70].

Moreover, 8-PN exhibited some other noteworthy biological activities potentially applicable for therapeutic purposes. In an assessment of its antidiabetic properties in mice, 8-PN prevented body weight gain, and improved insulin resistance and glucose tolerance [71]. 8-PN is also available as a constituent of nutritional supplements for breast enhancement. However, no clinical assessments to confirm this effect have been conducted [72]. A regular intake of phytoestrogen-rich foods is associated with a lower risk of cardiovascular diseases [31]. Additionally, 8-PN inhibited platelet aggregation and adhesion in an ER-independent manner, by the inhibition of enzymes and factors involved in coagulation [73]. Anti-atherosclerotic effects were also evaluated in a clinical study where 157 post-menopausal women were administered a herbal preparation containing a mixture of isoflavonoid-rich plants, hop cone powder, and garlic powder for 12 months. The intake of the herbal preparation had a positive effect on the thickness of the intima media of the common carotid arteries, and inhibited the growth of atherosclerotic plaques [74].

## 7. Safety Issues

Although natural-based preparations are generally considered to be safe, the same investigations as for chemical drugs are necessary to evaluate the overall safety and risks of the occurrence of side effects related to the use of hop phytoestrogens. In general, interactions with ERs and other mechanisms of action of phytoestrogens are considered to be a potential threat, due to possible carcinogenicity, and some authors claim that the evidence for its safe therapeutic use is insufficient [75,76].

In an experiment with ovariectomized rats that received 8-PN subcutaneously for 3 days (15 mg/kg of body weight), a significant increase in uterine wet weight and increased proliferation in the vaginal epithelium was found, possibly representing a potential risk for application in humans [77]. Increased proliferation was also observed in the MCF-7 breast cancer cell line as a result of the application of 8-PN. Furthermore, 8-PN induced proliferation in ovariectomized rat mammary glands after three days exposure, raising a question regarding the cancer-promoting effects of 8-PN in certain tissues [78]. However, in a following study, the application of a standardized hop extract did not stimulate mammary gland tumor growth [79]. Additionally, during a study focused on the evaluation of anti-osteoporotic activities, induction of proliferation in the uterus and endometrium by 8-PN was negligible compared to estradiol doses necessary to accomplish the same bone-protective outcomes. However, the authors admit that the extrapolation of results from rat experiments to humans is problematic, due to differences is pharmacokinetic parameters [66]. Moreover, another study, focused on the levels of prenylflavonoids achieved in breast tissue after low dose oral exposure, concluded that the concentration of 8-PN was too low to exhibit any ER-mediated actions that could contribute to carcinogenesis [80].

Other safety issues relate to low specificity and broad differences in the effect, depending on inter-individual variations [81]. Due to possible effects on hormonal regulation, it is advised that premenopausal women should avoid using nutritional supplements containing phytoestrogens intended e.g., for breast enhancements or relief of premenstrual symptoms [82]. Furthermore, where a particular pharmaceutical preparation contains a hop extract, the individual components might interact with each other and act as mutual antagonists, which could be a cause of adverse or unpredictable effects [83].

Aromatase inhibitors used for the treatment of breast cancer could interact with phytoestrogens, which brings up another safety concern. Breast cancer therapy is associated with the occurrence of menopausal symptoms as a result of induced depletion of estrogens by the anticancer drugs. Many patients are inclined to use nutritional supplements containing phytoestrogens to relieve this effect. It has been shown in breast cancer models in vitro, and in ER assays, that the proliferation of tumor cells was induced after the application of 8-PN. While genistein and commercially available nutritional supplements containing phytoestrogens increased the activity of aromatase, 8-PN acted as a strong inhibitor. Despite that fact, this study indicates that the use of phytoestrogens to relieve hormone-dependent side effects of breast cancer therapy should be avoided [16].

In addition, 8-PN affected the maturation of porcine oocytes in vitro, suggesting another possible risk for the use of prenylflavonoids in dietary supplements [84]. 8-PN also has the potential to inhibit cytochrome P450 (CYP450), specifically the CYP1A2, CYP2C9, and CYP2C19 subfamilies, which could affect the turnover of other substrates when administered simultaneously [85]. Common substrates of these CYP450 subtypes are caffeine, an anticoagulant drug warfarin, or omeprazole, used for the treatment of peptic ulcers, respectively [86]. The occurrence of side-effects of such therapeutics could be increased due to the effect of 8-PN on the enzymes [85]. Further assessment is necessary to precisely evaluate the safety data.

## 8. Conclusions

We have reviewed the interaction of 8-PN with ERs for potential use in the therapy of several hormone-dependent diseases. Our thorough overview of the literature has identified a number of trends. Firstly, clinical trials reported to date show that 8-PN in the form of a standardized hop extract has the ability to alleviate menopausal symptoms. Secondly, cell culture models have demonstrated that 8-PN is able to prevent osteoporosis by promoting the differentiation of osteoblasts and inhibiting the activity of osteoclasts. Thirdly, some anticancer activity has been confirmed in vitro. Furthermore, no adverse effects related to the application of 8-PN were observed in any of the clinical studies. That being said, some authors highlight concerns that 8-PN’s estrogenic mechanism of action may lead to undesired effects after long-term supplementation. Thus, additional pharmacokinetic studies are needed to further elucidate dose–response relationships with the aim of identifying an optimal dosing regime. In parallel, more biotransformation studies are required for the quantification and elimination of safety risks.
